# Supplementary material for: Natural Patterns in the Dawn and Dusk Choruses of a Neotropical Songbird in Relation to an Urban Sound Environment
Source: Animals (Basel). 2024 Feb 17;14(4):646. doi: 10.3390/ani14040646 (PMC10886165; doi:10.3390/ani14040646)
Supplement: Supplementary file 1 [file animals-14-00646-s001.zip › animals-2816722-supplementary.pdf]

SUPPLEMENTARY MATERIAL

Table S1. Specific information for each study plot with the parameters of the tracks recorded in each period of the day per plot (a.m. = morning; p.m. = afternoon).

Plot Code	Plot Name (mean dBA*) (Central point coordinates)	Period of the day	Number of recorded individuals of the Chiguanco Thrush	Total songs recorded	Total songs analyzed
U1	San Pedro (75 dBA)	a.m.	13	297	51
	(-16.502466; -68.135261)	p.m.	12	175	39
U2	Sopocachi (70 dBA)	a.m.	18	419	33
	(-16.514340; -68.128479)	p.m.	16	170	19
U3	Miraflores (75 dBA)	a.m.	15	216	29
	(-16.499655; -68.121093)	p.m.	17	145	28
E1	Auquisamaña (55 dBA)	a.m.	14	627	54
	(-16.554830; -68.071654)	p.m.	14	419	29
E2	Achumani (50 dBA)	a.m.	18	903	57
	(-16.510743; -68.047747)	p.m.	16	397	28
E3	Cota Cota (60 dBA)	a.m.	17	982	51
	(-16.538624; -68.069034)	p.m.	17	320	16

(*) Information obtained from the isophonic curves constructed with the mean values of measurements of sound pressure level obtained as dBA between 08:00 and 20:00 in the city of La Paz [50].

Table S2. Spearman's rank correlation values and *p*-value (in parenthesis), for pair-to-pair comparisons of the environmental sound pressure level (EPS) and the Chiguanco Thrush song variables: song sound pressure level (SSP), minimum song frequency (Min. F.), maximum song frequency (Max. F.), frequency range (F. Range) and dominant song frequency (Dom. F), for the urban zone in the upper diagonal and for the extra-urban zone in the lower diagonal of the city of La Paz.

	ESP	SSP	Min. F.	Max. F.	F. Range	Dom. F.
**ESP**		0.040<br>(= 0.577)	0.035<br>(= 0.619)	- 0.061<br>(= 0.395)	- 0.061<br>(= 0.390)	0.021<br>(= 0.801)
**SSP**	- 0.379<br>(< 0.001)		- 0.220<br>(= 0.002)	0.279<br>(< 0.001)	0.315<br>(< 0.001)	0.007<br>(= 0.934)
**Min. F**	0.370<br>(< 0.001)	- 0.303<br>(< 0.001)		- 0.132<br>(= 0.062)	- 0.324<br>(< 0.001)	- 0.042<br>(= 0.621)
**Max. F**	- 0.268<br>(< 0.001)	0.425<br>(< 0.001)	- 0.207<br>(= 0.001)		0.973<br>(< 0.001)	- 0.025<br>(= 0.770)
**F. Range**	- 0.312<br>(< 0.001)	0.454<br>(< 0.001)	- 0.391<br>(< 0.001)	0.974<br>(< 0.001)		- 0.014<br>(= 0.865)
**Dom. F.**	0.135<br>(= 0.057)	0.066<br>(= 0.356)	0.115<br>(= 0.106)	0.034<br>(= 0.630)	0.001<br>(= 0.991)	
